# Editorial: Microbial Electron Transport

**DOI:** 10.1093/femsec/fiaf103

**Published:** 2025-10-14

**Authors:** Lucian C Staicu, Catarina M Paquete

**Affiliations:** Faculty of Biology, Institute of Microbiology, University of Warsaw, Miecznikowa 1, 02-096 Warsaw, Poland; Instituto de Tecnologia Química e Biológica António Xavier, Universidade Nova de Lisboa, Av. da República Apartado 127, 2780-157 Oeiras, Portugal

**Keywords:** editorial, microbial electron transport, thematic issue

Electron transport is fundamental for all living organisms to generate the cellular energy needed to sustain growth. Microorganisms display an exceptional versatility in their use of electron donors and acceptors, including metals and metalloids, making them key players in Earth’s biogeochemical cycles (Staicu and Barton 2024). In recent decades, microbes capable of extracellular electron transfer have captured growing scientific and industrial interest. Their unique ability to exchange electrons beyond the cell membrane opens exciting opportunities in bioremediation, microbial electrochemical technologies, and the sustainable production of biofuels and chemicals. The thematic issue of FEMS Microbiology Ecology, entitled “Microbial Electron Transport”, brings together one review and seven research articles that collectively advance our understanding of microbial respiration and electron transport across environmental and applied contexts.

Environmental variability often determines the growth and activity of microorganisms. Bottaro et al. (2025) investigate how salinity and pH influence microbial communities capable of coupling nitrate reduction with Fe(II) oxidation. Using microcosm and enrichment experiments, they demonstrate that microbes can adapt to pH and salt fluctuations, with pH emerging as a key control factor. Their findings reveal that Fe(III) mineral precipitation is favored at neutral pH, shedding light on the intricate balance between environmental chemistry and microbial metabolism. In a different study, Soares et al. (2025) examine microbial community structure dynamics with Fe- and S-rich lithology in a pyritic coal-based aquifer. Their study shows that Fe(II)-oxidizing bacteria from the *Gallionella* and *Sideroxydans* genera dominate anthracitic and low volatile coal ranks, whereas S-oxidizing bacteria, such as *Sulfuricurvum* and *Sulfurovum* genera, prevail in medium volatile coal ranks. These results underscore the importance of hydrogeological and geochemical conditions in shaping microbial communities and regional Fe- and S-biogeochemistry. Cultivating chemolithotrophic neutrophilic Fe-oxidizing bacteria remains notoriously difficult due to the challenge of replicating their native environments in the laboratory. Addressing this limitation, Uchijima et al. (2025) introduce a custom-designed growth medium that simulates the energy substrates and nutrients typical of natural Fe-rich habitats. This innovation successfully enriches members of the *Gallionellaceae* family, opening new avenues for studying their metabolism and respiratory processes.

The interplay between Fe(II) oxidation and Fe(III) reduction is a defining feature of many aquatic systems. Nikeleit et al. (2025) explore these dynamics in a dimictic lake, revealing that potential Fe-metabolizing bacteria, including *Chlorobium, Thiodictyon, Sideroxydans, Geobacter*, and *Rhodoferrax* thrive predominantly under anoxic conditions. Their work highlights how organic substrates and redox gradients control the balance of iron cycling in natural waters.

The metabolic potential of *Thiobacillus denitrificans* as a nitrate-reducing Fe(II) oxidizer remains an open question. Toward this, Becker et al. (2025) approach this puzzle through multiple transfer cultivation experiments, using Fe(II) and nitrate as the sole electron donor and acceptor, respectively. Their findings suggest that *T. denitrificans* can perform the first step of denitrification using Fe(II) as an electron donor, yet it fails to sustain growth under these conditions.

A deeper understanding of microbial electron transport and its link to mineral transformations is vital for advancing biotechnological processes. Staicu et al. (2025) review how microbial interactions with biominerals, such as FeS_x_, S^0^, AsS, and Se^0^ influence both basic biological functions and applied processes. Their analysis bridges fundamental and applied perspectives, discussing implications for bioelectrochemical systems and highlighting the promise of this multidisciplinary field.

Microbial electron exchange is not limited to single species, as it also drives syntrophic cooperation. Feliu-Paradeda et al. (2025) demonstrate that co-cultures of two *Clostridium* species grown in the presence of conductive materials exhibited enhanced microbial interactions, boosting the production of elongated short-chain fatty acids and alcohols. These findings underscore the potential of conductive materials to mediate electron transfer between microbes, implying biochemical yields. In another contribution, Boltz and Rittmann (2025) explore autotrophic and heterotrophic bacteria in microbial communities based on H_2_-autotrophy. Through simulations under varying solids retention times, they revealed that autotrophic hydrogenotrophs dominate the active biomass, while non-active solids accumulate over time, providing insights into the stability and efficiency of mixed microbial systems.

Collectively, the studies presented in this thematic issue highlight that microbial electron transport is not only a cornerstone of metabolism but also a driver for ecological and technological innovation. We have high hopes that the corpus of work reported in this thematic issue will inspire further research on the multifaceted aspects of microbial electron transfer, from iron cycling in natural waters to engineering co-cultures for sustainable production, ultimately advancing our ability to harness these processes to meet pressing societal and environmental challenges.
